# Career Development Support, Job Adaptation, and Withdrawal Intention of Expatriates: A Multilevel Analysis of Environmental Factors

**DOI:** 10.3390/ijerph16203880

**Published:** 2019-10-13

**Authors:** Hemin Song, Shuai Zhao, Wenwen Zhao, Hua Han

**Affiliations:** 1 Sports Business School, Beijing Sport University, Beijing 100084, China; hanhua@bsu.edu.cn; 2 College of Information Engineering, Shandong Yingcai University, Jinan 250104, China; tony500perfect@163.com; 3 School of Management, Xi’an Jiaotong University, Xi’an 710049, China; zhaowenwen0405@xjtu.edu.cn

**Keywords:** environmental factors, career development support, job adaptation, withdrawal intention

## Abstract

The present study aims to explore the impact of career development support on job adaptation and withdrawal intention, and the multilevel moderating role of host country environmental factors. Through the questionnaire survey, we collected 242 expatriates’ data of 25 countries from China’s multinational corporations. Based on the constructed multilevel analysis model, we find: (1) career development support has a significant impact on job adaptation and withdrawal intention of expatriates; (2) job adaptation plays a mediating role between career development support and withdrawal intention; and (3) host country environment plays the multilevel moderating role between career development support and job adaptation. Through the multilevel model of host country environment, this study explores the mechanism of how career development support affects job adaptation and withdrawal intention. The conclusions enhance the understanding of the adaptation of expatriates and have important theoretical and practical reference value to achieve successful expatriate in the context of host country environment.

## 1. Introduction

Over the past decade, multinational corporations in emerging economies have made extraordinary achievements and gradually changed the global competitive landscape with the deepening of economic globalization. In addition, expatriates play an important role in international operations. By sharing the culture, work-related knowledge, and related management practice of the parent corporation with the host country, knowledge transfer [1,2,3], control [4], and coordination [5] are achieved, and expatriates lay a solid foundation for establishing the competitive advantage for multinational corporations. Therefore, it is of great practical significance to improve the adaptability of expatriates and reduce the withdrawal intention of expatriates to achieve successful expatriation. Scholars have proposed many corresponding measures for this point, such as improving the frequency of cross-cultural training for expatriates [6], maintaining favorable HCN-expatriate relationship [7] and enhancing the cultural intelligence of expatriates [8]. What cannot be ignored is that the impact of mentoring on expatriates has become an important topic in the research field of expatriate management [9]. Mentoring refers to an interpersonal exchange relationship [10] between older, more experienced, and knowledgeable employees (mentors) and young employees (apprentices) lacking in experience and knowledge. Especially when expatriates first enter their host country, the career development support under mentoring plays an important role. A specific type of mentor would meet each need from expatriates during the international assignment [11], and addresses occupational and psychological stress [12]. However, although scholars have made fruitful achievements in the impact of career development support under mentoring on expatriates, the conclusions and research objects are mostly based on the expatriates from multinational corporations of western developed countries, while there are few studies on multinational corporations from emerging economies, especially expatriates from Chinese multinational corporations [13]. Thus, the applicability of relevant conclusions to Chinese expatriates needs to be further analyzed. At the same time, as an overseas representative of a multinational corporation, expatriates have to face the uncertainty of local environmental factors (living conditions, medical and sanitary conditions, and public security situation of the host country), which poses challenges to the physical and psychological health of expatriates [14], aggravates problems of control within multinational corporations making it difficult to monitor expatriates’ behavior [15]. Especially the public security has gradually gained widespread attention with the prevalence of global terrorism activities in recent years [16]. Unfortunately, few studies have examined the impact of host country environment on expatriates. According to the statistics of the Ministry of Commerce, as of the end of 2017, 25,500 domestic investors in China had established a total of 39,200 overseas corporations, distributed in 189 countries and regions, with 1.7 million expatriates, accounting for 50% of all expatriates. Therefore, it is important to study the expatriates from Chinese multinational companies. Existing research has shown that the impact of career development support on apprentices is double-edged, with both positive and negative impacts [17,18]. For expatriates, the role of career development support from mentors may be more complicated. Some scholars argue that it has little impact on reducing the work pressure or improving organizational identification and job satisfaction of expatriates [19]. Thus, it needs to be further explored whether career development support can play its due role under the impact of the host country environment for expatriates in Chinese multinational corporations. Therefore, this research aimed to provide a theoretical reference for successful expatriate by constructing a multilevel moderating model to analyze the impact of career development support on the job adaptation and withdrawal intention of expatriates in the context of their host country.

## 2. Theories and Research Hypotheses

### 2.1. Mentoring Based on Social Exchange Theory

The traditional view is that “mentors” refer to a higher-level individual who provides guidance and assistance for subordinates so as to improve their ability [20,21]. For expatriates, individuals who can act as “mentors” are usually divided into three categories: superiors, colleagues at home and abroad, and others. It is worth noting that citizens in the host country can also act as “mentors,” even if they are not in a superior–subordinate relationship with the expatriates. According to Scandura [22], mentors can provide guidance for apprentices from three aspects: career development, social psychology, and role model. Career development guidance mainly includes professional and workplace skills; social-psychological guidance includes counseling and care; and role model guidance mainly includes role positioning [12].

Social exchange theory assumes that the goal of individuals in social relations is benefit maximization and cost minimization [23]. The benefits in social exchange mainly include the fun, friendship, information, and support obtained from the social relationships, while the individual costs invested in the social relationships include mostly money, time, and effort. In fact, both sides of the social exchange are often attracted to each other because of internal and external remuneration. Internal remuneration includes love, honor, and social status, while external remuneration includes potential compensation, services, and commodities. One of the essential principles of social exchange theory is the principle of reciprocity, which means that both sides should give and obtain benefits fairly to ensure the balance of social exchange. In addition, the realization of social exchange depends on the action of the other side, i.e., the support of one side depends on that of the other. Obviously, the career development support under mentoring is related to money, social status, information, and services. Therefore, mentoring reflects a special form of social exchange relationship, namely the hierarchical relationship. Under this hierarchical relationship, “mentors” have a rich experience, more resources and higher status, while “apprentices” are often lacking in skills and relevant experience. Based on social exchange theory, both mentors and apprentices should benefit from mentoring; not only does it enable apprentices to have higher professional expectation and salary level, improve their job satisfaction and retention wishes, and benefit their career advancement, but it also enables “mentors” to obtain inner respect and satisfaction and improves their job performance [24].

### 2.2. Career Development Support and Job Adaptation of Expatriates

The career development support under mentoring, as the most relevant dimension of expatriates, has an important impact on the job adaptation of expatriates. Based on social exchange theory, “mentors” provide career development support to expatriates so that they can further clarify their roles in the work, gain job-related skills, reduce uncertainty and pressure of work, and accelerate the socialization process of expatriates, thereby improving their job adaptation.

Social exchange theory assumes that both sides have the goal of obtaining a return. In many cases, friends and citizens in the host country who act as “mentors” do not require a return, thus there are some limitations to studying the role of mentoring via social exchange theory. However, an important development of social exchange theory is introducing relevant concepts of the social network into social exchange relationships so that the relationships among social exchange, social network and social capital become closer, thus laying the foundation for studying the impact of mentoring on the job adaptation of expatriates. It is generally accepted that one’s social network refers to a collection of people with one or more types of interpersonal relationships, while social capital represents the sum of real and potential resources embedded in the relationship network owned by individuals or social groups [25]. The social network usually includes two types of relationships: emotional relationships and instrumental relationships. Instrumental relationships mainly include collecting necessary information, suggestions, and resources for accomplishing work tasks, which is strongly related to career development support.

There are five main characteristics of the social network of expatriates: scale, diversity, localization, closeness, and frequency [26]. Scale refers to the number of expatriate colleagues, local colleagues and friends; diversity describes the source of actors in the personal network of expatriates; localization refers to the supporting degree of citizens in the host country for expatriates; closeness refers to the contact of expatriates with network members; and frequency refers to the number of times that expatriates contact the network members in the unit time. The characteristics of social network can enhance the explanatory power of the impact of mentoring on the job adaptation of expatriates. In the context of the host country, “mentors” of expatriates usually have the characteristics of “multiplicity”. The number and source of individuals who act as “mentors” are increasing and the social network of expatriates is also enlarging. Expatriates are better able to obtain comprehensive career development support from many “mentors,” thus improving their job adaptation. The closer is the relationship between expatriates and “mentors” and the more frequent they communicate with each other, the easier it will be for expatriates to learn relevant work skills and maintain good working relationships, thus improving their job adaptation. Therefore, this research proposes the following hypothesis:

**Hypothesis** **1.** *The career development support of mentoring exerts a significant positive impact on the job adaptation of expatriates.*

### 2.3. Career Development Support and Withdrawal Intention of Expatriates

Withdrawal intention, prior to a series of withdrawal behaviors such as absenteeism and dismissal [27,28], usually consists of three dimensions: the intention to abandon work tasks, leave the company, and give up the career. The intention to abandon work tasks describes employees’ intention to leave the current job, but the scope is limited to the company; the intention to leave the company means that employees select other companies; and the intention to give up the career mainly describes employees’ intention to pursue different careers in the future. Different from the withdrawal intention, the intention to leave the company usually means that individuals leave the organization. Due to the particularity of the expatriate, the concept of withdrawal intention can more accurately describe the complexity of expatriates. For expatriates, their withdrawal intention includes the intention to leave their current country, to leave their company, and to abandon their current career.

Based on social exchange theory, the career development support of mentoring can not only improve the job skills of expatriates, but also reduce (or shifts) the psychological pressure of expatriates. Meanwhile, it improves the familiarity of expatriates with work tasks and enhances the confidence of expatriates in successfully completing their time in their host country. As a result, it will reduce the probability of failure of the expatriation and enhance their willingness to stay in their position. From the perspective of the social network, these supports enhance the return intention of expatriates to their “mentors” as well as the friendship between expatriates and “mentors”. In addition, it helps to maintain a stable and good social network relationship. Thus, they are more willing to accept further guidance from their “mentors” in the future and to maintain frequent communication and close contact with their “mentors”. In many cases, “mentors” are appointed by subsidiaries to provide guidance for expatriates, thus the return intention and emotional commitment of expatriates to their “mentors” often shift to the loyalty, organizational commitment, and recognition of the overseas subsidiary, thereby reducing the withdrawal intention of expatriates. Therefore, this research proposes the following hypothesis:

**Hypothesis** **2.** *The career development support of mentoring exerts a significant negative impact on the withdrawal intention of expatriates.*

### 2.4. Job Adaptation and Withdrawal Intention of Expatriates

The inadaptability of expatriates reflects that they are lacking in appropriate disposing capacity in the context of the host country [29], which may increase the probability of expatriates making mistakes. To avoid further deterioration of this adverse effect, expatriates often have a strong withdrawal intention. Expatriates with strong job adaptation will not have great work pressure when facing the challenges from their host country, thus they can be more concentrated on their tasks. With the extension of expatriate tasks, the recognition and loyalty of expatriates toward their subsidiaries will be enhanced, thereby increasing their retention intention and reducing their withdrawal intention. In fact, the relationship between the job adaptation and withdrawal intention of expatriates can be explained based on cognitive dissonance theory [30]. It is argued in cognitive dissonance theory that, in the same scope, when the cognition of an individual is inconsistent with others, cognitive dissonance will occur, and the individuals will feel tension and pressure. This pressure drives individuals to change this cognitive difference to reduce cognitive dissonance. For example, within the subsidiary, expatriates may consider themselves to be good managers. However, when subordinates or other colleagues in the host country have a negative evaluation of their management ability, it violates the expatriates’ self-image. Under this cognitive dissonance, expatriates cannot adapt to the culture and working environment of the host country and they can only choose to withdraw from their expatriation to address this situation. Therefore, the job adaptation of expatriates has a negative impact on withdrawal intention of expatriates.

From the perspective of pressure theory, pressure is generated by the interaction between individuals and environmental factors [31]. When expatriates feel huge pressure due to other factors, they feel they cannot adapt to their work, thus they will have a negative attitude toward their expatriate tasks, invest less energy in expatriate tasks, have stronger job burnout and lack appropriate disposing capacity. Even with strong career development support, under the interference of external factors, this huge work pressure often leads to poor adaptability of expatriates, and they still have strong withdrawal intention. Therefore, the job adaptation of expatriates has become an important part of career development support to reduce withdrawal intention. Therefore, the job adaptation of expatriates plays a moderating role (partial mediating effect) between career development support and withdrawal intention. Based on this, the research proposes the following hypotheses:

**Hypothesis** **3.** *The job adaptation of expatriates exerts a significant negative impact on their withdrawal intention.*

**Hypothesis** **4.** *The job adaptation of expatriates plays a moderating role in career development support and withdrawal intention.*

### 2.5. Multilevel Moderating Effect of the Host Country Environment

The social environment usually refers to the social system formed by intertwined biological genetic factors, psychological states, and social processes that affect human behavior. Environmental behavior theory argues that the environmental stimulus will lead to the physiological and psychological effect of human beings and this effect on human beings will reflect in extrinsic behaviors. When environmental factors cannot fully satisfy people’s needs, their behavior will be restricted by the environment. The host country environment, as an important part of the social environment, has an important impact on expatriates, which mainly includes the living conditions, medical conditions, food quality, and public security status of the host country. Different host countries have different environments, thus exerting different impacts on expatriates. For example, compared with developed countries, the relatively harsh environment in developing countries reduces the comfort and satisfaction of expatriates [14], thus reducing the job adaptation of expatriates. In fact, the uncertainty of the host country environment is often regarded as a source of pressure for expatriates [32] and this pressure is often closely related to the adaptation of expatriates.

Based on trait activation theory, the role of the host country environment in career development support and the adaptation of expatriates become subtler. Personality traits usually describe the tendency to be consistent within individuals and unique among individuals, and this tendency can be expressed in an identifiable manner [33]. A personality trait is a potential construct that can only be activated in certain situations. Therefore, trait activation theory believes that trait activation refers to the process of expressing traits when individuals face trait-related situational triggers [34]. In other words, the behavioral reflection of a trait needs to be awakened by trait-related situational triggers. To understand under what circumstances a personality trait can be expressed through behavior, trait activation theory emphasizes the importance of situational trait association. If one certain situation can provide triggers for trait-related behavioral expression, then this situation is considered to be related to traits. In the research field of management, trait activation theory can explain the difference when using certain traits in predicting job performance in different work contexts. For expatriates, the host country environment can enhance trait-related situational triggers, awaken related traits of expatriates and produce corresponding behavioral expression. Therefore, the use of trait activation theory can explain the impact of host country environmental effects on expatriates.

In the context of the host country, a better environment implies better environmental support, enhances the significance of triggers related to the personality trait of expatriates, activates the traits required by expatriates to successfully complete the expatriate, and inspires the working enthusiasm of expatriates, thus achieving positive expatriate effects. Although a poor host country environment can also arouse personality traits to a certain extent, expatriates need to make greater efforts to overcome a poor host country environment, thereby enhancing the relationship between the career development support and job adaptation of expatriates. However, a poor host country environment means the lack of many conditional supports and expatriates often have negative psychological feelings such as pessimism. Even with higher level of career development support, expatriates will still feel that their ability cannot satisfy the requirement of expatriate tasks, thus reducing the relationship between career development support and job adaptation of expatriates. Therefore, from the perspective of trait activation theory, the host country environment can regulate the relationship between career development support and job adaptation of expatriates. This regulating effect is often reflected in the fact that the better is the host country environment, and the more the career development support adapts to the work of the expatriate, the stronger is the positive relationship between career development support and job adaptation of expatriates. Therefore, this research proposed the following hypothesis:

**Hypothesis** **5.** *The host country environment regulates the relationship between the career development support and job adaptation of expatriates in a positive manner. The better is the host country environment, the stronger is the impact of career development support on job adaptation.*

**Figure 1:**
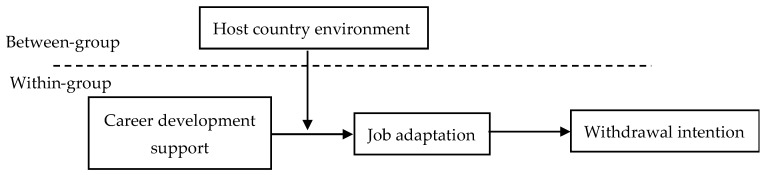
Conceptual model.

Therefore, this article constructed the conceptual model (Figure 1) according to the above hypotheses. In this model, career development support is regarded as an independent variable, withdrawal intention is a dependent variable, job adaptation plays a mediating role at the individual level, and host country environment plays a moderating role at the organizational level between career development support and job adaptation. 

## 3. Research Methodology

### 3.1. Research Sample

This research selected multinational corporations on the list of the websites of China’s Ministry of Commerce and distributes questionnaires via online links to related corporations for expatriates to fill out. In total, 345 questionnaires were collected before the deadline, of which 242 were valid (from 25 countries and regions, Table 1), with the effectiveness rate of 70%. According to the recovery of questionnaires, the number of males was 187 (accounting for 77%) while the number of females was 55, (23%); the age of expatriates was between 20 and 55 years old, with an average age of 32; the number of people who received cross-cultural training before expatriation was 141 (58%), while the figure of not receiving cross-cultural training was 101 (42%); the average overseas working experience before expatriation was 2.4 years, of which 101 people had at least three years of overseas working experience (42%) while 141 people who had less than three years of overseas working experience (58%).

### 3.2. Measurement of Variables

This study used the research scale of career development support, host country environment, job adaptation, and withdrawal intention under mentoring in existing domestic and foreign literature. The scale was appropriately modified by two-way translation, and the questionnaire was finally formed. The specific scale sources were as follows:

(1) Career development support under mentoring:

This study drew on the research scale of Scandura and Schriesheim [35] and selected the career development support dimension (four measurement items) provided by mentors for expatriates. The measure was conducted using a seven-point Likert scale (from “strongly disagree” to “strongly agree”).

(2) Host country environment:

With regard to the measurement of the host country environment, this study drew on the research scale of Birdseye and Hill [14], which mainly describes expatriates’ evaluation of the host country environment, including seven measurement items. The measurement items were conducted using a five-point Likert scale (from “strongly dissatisfied” to “strongly satisfied”).

(3) Job adaptation:

This study drew on the research scale of Black and Stephens [32], which mainly includes the adaptation to specific job responsibilities, performance standards, and regulatory responsibilities, including three measurement items. The measurement was conducted using a five-point Likert scale, with 1 indicating very poor, 2 poor, 3 neutral, 4 good, and 5 very good.

(4) Withdrawal intention:

This study drew on the research scale of Shaffer et al. [36], which mainly describes the withdrawal intention of expatriates from the current expatriate task, including four measurement items. The measure was conducted using a five-point Likert scale (from “strongly disagree” to “strongly agree”).

(5) Control variables:

It is necessary to consider the role of control variables in the process of studying the relationship between mentoring, job adaptation, and withdrawal intention, including age [37], previous internationalization experience [38], cross-cultural training before the expatriate [39], and language proficiency of host country [40]. Regarding the cross-cultural training before the expatriate, this study used dummy variables to explore whether expatriates received the cross-cultural training; for the language proficiency of host country, this study used a five-point Likert scale for the measurement. In the scale, the options were “very poor”, “poor”, “general”, “skilled”, and “proficient.”

### 3.3. Common Method Variance and Testing of Validity and Reliability

Since the questionnaire scale was self-reported, there may be a problem of common method variance. This study used the Harman single-factor test to assess the problem of common method variance [41]. The unrotated principal component factor analysis showed that the variation of the interpretation of the first factor was 33%, which is not beyond the critical value of 40%, indicating that the problem of common method variance was not serious, thus the reliability and validity of the scale could be further tested.

Regarding the test of reliability and validity, the Cronbach’s α coefficient of job adaptation was 0.782, and the constructed reliability value (CR = 0.795) and the average refining variance (AVE = 0.573) both reached the desired effect, indicating good convergence validity. The Cronbach’s α coefficient of withdrawal intention was 0.842, and the constructed reliability value (CR = 0.846) and the average refining variance (AVE = 0.583) both reached the desired effect, indicating good convergence validity. The Cronbach’s α coefficient of career development support under the mentoring was 0.861, and the constructed reliability value (CR = 0.863) and the average refining variance (AVE = 0.614) both reached the desired effect, indicating good convergence validity. In addition, it showed good fitness (CFI = 0.980, GFI = 0.979, RMR = 0.037, and SRMR = 0.03). The perceived Cronbach’s α coefficient of the host country environment was 0.879, and the constructed reliability value (CR = 0.882) and the average refining variance (AVE = 0.520) both reached the desired effect, indicating good convergence validity. In addition, it showed good fitness (CFI = 0.945, GFI = 0.938, RMR = 0.045, and SRMR = 0.04). Finally, the integration of latent variables reveals that the discriminatory validity of the four-factor model was better than other factor models (Table 2).

### 3.4. Data Aggregation and Analysis Methods

The host country environment emphasizes expatriates face the environment of different countries, which is the concept of the organizational hierarchy. Firstly, this study obtained the perceived host country environmental scale via expatriates’ assessment toward the host country environment. Secondly, the data of the individual level of expatriates were aggregated to the organizational level. Since the analysis involved two levels of individuals and organizations, this study used hierarchical linear modeling (HLM) for the analysis and it was discovered that there was a significant difference in terms of the between-group variance and within-group variance of the perceived host country environment (F = 225.91, *p* < 0.001). The ICC (1) and ICC (2) were 0.446 and 0.82, respectively, which met the aggregation requirements. The median of the perceived host country environment *Rwg* was 0.93, which was consistent with the basic standard of within-group assessment consistency. Therefore, it was appropriate and effective to aggregate the perceived host country environment at the organizational level.

## 4. Multilevel Model Test

### 4.1. Descriptive Statistics and Correlation Analysis

Table 3 shows the mean, standard deviation, and correlation coefficient of relevant variables. It can be seen in the Table that there was a positive correlation between the career development support and job adaptation of expatriates, and a negative correlation with withdrawal intention; there was a negative correlation between job adaptation and withdrawal intention; and there was a significant correlation between the perceived host country environment and job adaptation, withdrawal intention and career development support. The results of the correlation test could lay the foundation for the hypothesis testing of subsequent models.

### 4.2. Results of Multilevel Analysis

This study used the hierarchical linear modeling for data analysis, substituting the age, international experience, cross-cultural training, language proficiency, job adaptation, withdrawal intention, and career development support of expatriates into the individual level of HLM analysis (Level 1) and the host country environment into the organizational level (Level 2). Table 4 shows the results of cross-level regression analysis using HLM. Firstly, the variation of job adaptation was decomposed at the individual level and organizational level without introducing any explanatory variables to analyze the variation of job adaptation at the organizational level (Model 1). It was found that the inter-organization variation of job adaptation was significant (τ00 = 0.05, *p* < 0.001, ICC (1) = 0.14, and ICC (2) = 0.53), thus it was reasonable to perform a multilevel analysis. Secondly, according to the analysis procedure of Zhang et al. [42], when career development support was added to the job adaptation model (Model 2), career development support exerts a significant positive impact on job adaptation (γ = 0.18, *p* < 0.01) and thus H1 was verified; when career development support was added to the withdrawal intention model (Model 5), career development support exerts a significant negative impact on withdrawal intention (γ = −0.17, *p* < 0.01), and thus H2 was verified; and when the career development support and job adaptation were added into the withdrawal intention model (Model 6), both career development support (γ = −0.12, *p* < 0.05) and job adaptation (γ = −0.30, *p* < 0.01) exert a significant impact and thus H3 was verified. Combined with H1, job adaptation had a partial mediating effect between career development support and withdrawal intention (mediating effect accounting for 31%), thus H4 was verified.

**Figure 2:**
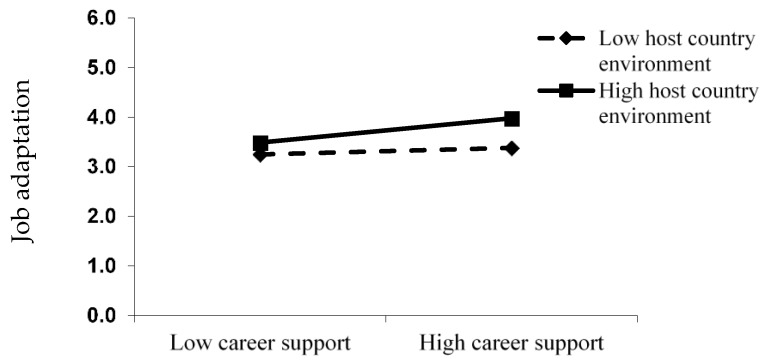
The moderating role of host country environment.

To verify the mediating role of host country environment between career development support and job adaptation, this study added the product term of career development support and host country environment into the job adaptation model (Model 3), and it was found that the coefficient of the product term is significant (γ = 0.15, *p* < 0.01). Compared with Model 2, τ00 decreased from 0.29 to 0.08, indicating that the product term of the career development support and host country explains 72.4% of the between-group variance of job adaptation. To verify the significance of the mediating effect, this study used the research method of Aiken and West [43] (Figure 2). In a poor host country environment, the positive impact of career development support on job adaptation was not significant (effect = 0.39, *p* > 0.10); on the contrary, in good host country environment, the positive impact of career development support on job adaptation was more significant (effect = 0.62, *p* < 0.01). Therefore, H5 was verified.

In addition, to test the regulated mediating effect model, this study used the Bootstrap method of parameters for the estimation with the help of R software (Table 5). The results show that, at the 95% significance level, in a poor host country environment, the indirect effect of career development support on withdrawal intention was significant (indirect effect = −0.16, 95% confidence intervals = [−0.29, −0.06]); while, in a good host country environment, the indirect effect of career development support was also significant (indirect effect= −0.23, 95% confidence intervals = [−0.43, −0.08]), and the between-group variance was also significant (difference = −0.07, 95% confidence intervals = [−0.14, −0.02]).

## 5. Conclusions

### 5.1. Research Conclusions

This study explored the relationships among career development support, job adaptation, and withdrawal intention under mentoring in the host country environment. The results show that career development support had a significant impact on job adaptation and withdrawal intention; the job adaptation of expatriates had a significant negative impact on the withdrawal intention and it played a partial mediating role between career development support and withdrawal intention; the host country environment had a regulating role between career development support and job adaptation and this regulating effect could only be achieved in a good host country environment. In the poor host country environment, the positive relationship between career development support and job adaptation was weakened.

(1) The direct effect of career development support under mentoring:

The significant impact of career development support indicates that “mentors” had a positive effect on improving the adaptation of expatriates and reducing their withdrawal intention in terms of job skills. Similar to the conclusions of most studies [11,12,44], the career development support of mentors was beneficial for expatriates to clarify their work roles and obtain information related to the dynamic change of work environment, thus reducing the working pressure, improving work adaptation, and reducing the withdrawal intention of expatriates. Although some scholars believe that a lower degree of support at the company level is beneficial to stimulate the personality traits of expatriates and generate positive effects based on the trait activation theory [45], if there is no clear description and basic demand information of the expatriate task, the trait activation may not be able to achieve the desired effect. Some studies argue that the career development support of mentors may reduce the job satisfaction of expatriates, especially when domestic mentors are lacking in relevant experience or the existing experience cannot be applied to the host country environment [19], thus generating strong withdrawal intention. However, multinational corporations can select specific mentors through environmental-experience matching to improve the guidance intensity for expatriates. In addition, many factors affect the withdrawal intention of employees, such as job insecurity [46], unsatisfactory salary [47], etc., thus, even with lower career development support, expatriates may still choose to retain in their posts because of the enhancement in internationalization experience or attraction of high pay, thus reducing their withdrawal intention. Of course, the positive role of career development support is still of certain explanatory power when excluding other factors.

(2) The complete mediating effect of the job adaptation of expatriates: 

This research found that the job adaptation of expatriates had a significant negative impact on the withdrawal intention and this conclusion was similar to most of the literature [27,48]. A few studies have shown that expatriate experience and attractive remuneration are important to their career [49], which means that expatriates may cherish expatriate opportunities, and thus low job adaptation does not necessarily lead to the withdrawal intention of expatriates. Conversely, when expatriates perceive the expatriate experience and remuneration as not attractive, even higher job adaptation may result in the withdrawal intention [27]. When excluding the interference effect of these factors, the positive impact of the job adaptation of expatriates is rational. Meanwhile, the impact of the career development support from “mentors” on the withdrawal intention of expatriates played part of its role through job adaptation. In other words, when “mentors” provide guidance to expatriates, they will obtain relevant skills and work information, thus improving their job adaptation, and indirectly reducing their withdrawal intention. Therefore, improving the job adaptation of expatriates is of great significance for overseas subsidiaries.

(3) Regulating effect of the host country environment:

Based on trait activation theory, this study examined the boundary conditions in which the career development support under mentoring affects the job adaptation under the regulating effect of host country environment. It was found that the regulating role could only be played in a good host country environment, while there was no regulating effect in a poor host country environment. In other words, in a poor host country environment, the impact of career development support under mentoring on the job adaptation was not significant. As with the analysis in Model 3, the impact of the host country environment might dilute the explanatory power of career development support, which verified the conclusions in some studies. Poor host country environment often leads to the dissatisfaction and discomfort of expatriates [50], and it is difficult to stimulate the enthusiasm and initiative of expatriates. Especially when there are public security problems such as terrorism in the host country, the working pressure and attitude toward the expatriate task will be negatively affected [16]. At this time, even with great career development support from “mentors,” it is difficult for the job adaptation of expatriates to achieve the desired effect. Only when the host country environment becomes better, expatriates can maintain a cheerful mood to be engaged in the expatriate task. At this time, the career development support from “mentors” can exert a positive impact. Therefore, how to make up for the disadvantages and improve the host country environment has become an important issue in the field of expatriate management.

### 5.2. Practical Significance

(1) Constructing an expatriate management system based on career development support:

The job adaptation of expatriates is an antecedent of the withdrawal intention and it is of great significance for Chinese multinational corporations to construct an expatriate management system to enhance the job adaptation of expatriates. First, the guidance for expatriates should be strengthened through the career development support of “mentors”. The “mentor” appointed by the corporation should use a customized guidance model based on the particularity of the host country to improve the quality of guidance. In addition to the guidance of working roles and skills, they should also provide further guidance for the language proficiency of the host country and evaluate the effectiveness of career development support. For expatriates accompanied by family members, the role of “mentors” should be extended to strengthen the training of expatriates and their family members in interpersonal, communication, and language skills, which can not only strengthen the adaptation of family members, but also create a good family–work match situation for expatriates. In addition, “mentors” should provide real-time guidance for expatriates regarding the strength and frequency of career development support. Although projects such as the cross-cultural training before the expatriate can play a certain role, expatriates are not exposed to real host country environment at this time, which means that this training cannot solve the accidents and challenges during the expatriate. Therefore, “mentors” appointed by foreign subsidiaries should provide real-time guidance to expatriates according to specific needs, and enhance or reduce the intensity of guidance according to specific environmental factors, so that expatriates can adapt to the living and working environment of the host country as soon as possible. Moreover, Chinese multinational corporations should reward mentors who contribute their time to inexperienced expatriates in the aspect of career development support in order to increase their motivation.

(2) Improving conditions to eliminate the negative effect of the host country environment:

This study verified the limitation of the role of career development support in a poor host country environment. For expatriate managers, on the one hand, the negative impact brought by poor host country environment can be alleviated by improving the necessary work and living conditions; on the other hand, other incentives can be used to make up for the disadvantages of host country environment. In addition to remuneration and family incentives, the interaction with citizens in the host country can be strengthened, which can reduce the loneliness and negative emotions of expatriates to a certain extent, stimulate the working enthusiasm of expatriates, and indirectly reduce the withdrawal intention while improving the job adaptability.

### 5.3. Research Insufficiency and Prospects

Although the study examined the applicability of the model through empirical analysis, there are still some limitations in this research:

(1) In terms of research method, the data were collected in the form of questionnaire filled out by expatriates, which may be subjective. Therefore, future research can be supplemented by the case study method and in-depth corporate interviews can be performed. In addition, related issues of expatriates can be investigated through single- vs. multi-case comparison to more clearly reflect the current situation of corporate expatriate management.

(2) In terms of research objects, the expatriates involved in this paper are mainly staff who are sent from Chinese corporations overseas. In fact, with the deepening of corporate internationalization, multinational corporations are increasingly concerned with the international operation based on localized employees and self-initiated expatriates [51]. Thus, the research on such subjects needs to be strengthened.

(3) In terms of the selection of variables, many variables are not included in the research framework, such as the emotional intelligence, cultural intelligence, and knowledge transfer of expatriates. It is expected that the research variables can be expanded in the future to perform an in-depth analysis of related management issues of expatriates.

## Figures and Tables

**Table 1:** Number of expatriates assigned to regions.

Region	Expatriates	Region	Expatriates
Papua New Guinea	4	Saudi Arabia	38
Brazil	3	Sri Lanka	7
East Africa	9	Thailand	2
Russia	4	Turkey	2
France	3	Venezuela	7
Philippines	6	Uzbekistan	34
Kazakhstan	14	West Africa	17
Cambodia	4	Hong Kong, China	4
Malaysia	6	Singapore	8
America	7	Iran	14
Mexico	3	India	5
Japan	2	Indonesia	33
England	6	Total	242

**Table 2:** Results of confirmatory factor analysis.

Models	χ^2^	*df*	χ^2^/*df*	RMSEA	RMR	SRMR	CFI
Four-factor	281.1	129	2.18	0.07	0.06	0.06	0.93
Three-factor	503.5	130	3.87	0.11	0.63	0.30	0.83
Two-factor	522.1	131	3.99	0.11	0.82	0.36	0.82
One-factor	545.6	132	4.13	0.11	1.09	0.41	0.81

**Table 3:** Descriptive statistics and correlation analysis.

Variables	1	2	3	4	5	6	7	8
Age								
Pre-experience	0.32 **							
Train	0.01	−0.01						
Language	0.04	0.24 **	0.08					
Job adaptation	−0.09	−0.03	0.24 **	0.11				
Withdrawal intention	−0.01	0.02	−0.07	0.03	−0.28 **			
Career support	−0.06	0.00	0.30 **	−0.03	0.41 **	−0.22 **		
Perceived host country environment	−0.00	0.06	0.23 **	0.11	0.53 **	−0.18 **	−0.41 **	
Samples	242	242	242	242	242	242	242	242
Mean	32.26	2.40	0.58	2.70	3.71	2.50	4.77	3.36
SD	5.62	2.69	0.49	1.11	0.60	0.77	1.03	0.77

Note: ** *p* < 0.01.

**Table 4:** Results of hierarchical linear model analysis.

Variables	Job Adaptation			Withdrawal Intention		
	Model 1	Model 2	Model 3	Model 4	Model 5	Model 6
Intercept	3.69 **	2.60 **	3.52 **	2.47 **	3.38 **	4.15 **
Level 1 main effects						
Age		−0.00	−0.00		−0.00	−0.00
Pre-experience		−0.01	−0.02 ^+^		0.00	0.00
Train		0.12	0.12		−0.05	−0.01
Language		0.01 **	0.08 **		0.01	0.04
Career support		0.18 **	0.15 **		−0.17 **	−0.12 *
Job adaptation						−0.30 **
Level 2 main effect						
Host country environment			0.36 **			
Cross-level interaction						
Career support × Host environment			0.15 **			
σ^2^	0.31	0.27	0.26	0.55	0.49	0.47
τ00	0.05	0.05	0.02	0.05	0.04	0.02
*Deviance*	424.2	410.0	405.0	555.4	558.9	549.5
*R^2^_within_*		0.11	0.22		0.12	0.18
*R^2^_between_*		0.05	0.41		0.17	0.37
*Pseudo R^2^*		0.10	0.23		0.12	0.18

Note: ** *p* < 0.01, * *p* < 0.05, and + *p* < 0.10.

**Table 5:** Results of the parameter bootstrapping test.

Moderated Mediation Model	Indirect Effect	95% LLCI	95% ULCI
Low host country environment	−0.16	−0.29	−0.06
High host country environment	−0.23	−0.43	−0.08
